# Tramadol half life is dose dependent in overdose

**DOI:** 10.1186/s40199-015-0104-y

**Published:** 2015-02-26

**Authors:** Hamid Khosrojerdi, Ghazal Alipour Talesh, Gholam Hassan Danaei, Sara Shokooh Saremi, Afrouz Adab, Reza Afshari

**Affiliations:** Addiction Research Centre, Imam Reza (p) Hospital, Ibn-e-Sina Street, Mashhad University of Medical Sciences, Mashhad, 9133316791 Iran; Nanotechnology Research Centre, School of Pharmacy, Mashhad University of Medical Sciences, Mashhad, Iran

**Keywords:** Tramadol, Toxicokinetic, Half-Life, Overdose

## Abstract

**Background:**

Tramalol overdose is disproportionately more common in Iran. In recent years, Tramadol overdose has become one of the most common causes of poisoning admissions to emergency departments in this country. To the best of our knowledge, there is little or no information regarding the toxicokinetic properties of Tramadol such as its half life. Given the fact that poisoning management should be based on the toxicokinetic of substances, we aimed at investigating the half life of Tramadol in man as a critical toxicokinetic variable in overdose.

**Methods:**

Blood samples of each patient were collected on admission and repeated later. Plasma was harvested after separation from blood cells by centrifugation and quantified using HPLC method. Calculations were performed on Tramadol blood concentration quantities.

**Findings:**

Demographic: Most of cases were men (81.81%). Mean (Standard Deviation (SD), min-max) age was 23 (8.142, 17-40). Serum Tramadol levels: Mean (SD, min-max) first Tramadol concentration was 786.91 (394.53, 391-1495). Mean (SD, min-max) second Tramadol concentration was 433.09 (269.63, 148-950). Mean (SD, min-max) of Tramadol half life was calculated as 9.24 hour (2.310, 4.99-13.45) Associations: Half life was associated with higher concentrations (r=0.708 Sig=0.015).

**Conclusion:**

We report the mean half life of tramadol in overdose to be 9.24 hours which is remarkably higher than that measured in previous pharmacokinetic studies. We also concluded that Tramadol half life is dose dependent in overdose which may explain the further consequences of severe overdoses.

## Findings

### Background

Tramadol is a synthetic, centrally acting analgesic opioid. It is mainly metabolized to *O*-desmethyl Tramadol (M1), which is also active [1]. Tramadol has been approved in some countries since 1980 and become the most frequently prescribed opioid around the globe [2]. Tramadol is rapidly and almost fully absorbed after single or multiple oral administrations. Nevertheless, the mean absolute bioavailability of Tramadol is reported to be 65–70% which is due to its extensive first-pass hepatic metabolism [3]. The bioavailability rises to >90% with multiple doses. The large volume of distribution (306 liters) after oral administration suggests its high tissue affinity [4]. Although frequently prescribed, few data on kinetic of Tramadol in humans are available. In a study performed on horses, half-life after intravenous and intramuscular administration was 82 ± 10 and 92 ± 14 min, respectively [5]. In the rat, the terminal elimination half-life of Tramadol is approximately 3 hours [6]. The distribution and elimination half-lives in humans were 1.02 and 141.9 min respectively [7].

Medicinal toxicities are common in Iran [8]. In recent years, Tramadol overdose has become one of the most common causes of poisoning admissions to emergency departments in this country [9,10]. The increasing number of Tramadol abuse and overdoses provokes the need for a more profound knowledge about the kinetic/dynamic profile of Tramadol in overdose. Several studies have been performed to measure the pharmacokinetic profile of this drug [11]. However, to the best of our knowledge, there is little information regarding the toxicokinetic properties of Tramadol such as its half life.

We previously reported the clinical manifestations of Tramadol overdose in relation to alleged dose [12]. The unique feature of this study is defining the half life of Tramadol as a critical toxicokinetic variable, in man in overdose.

### Method

This prospective cross-sectional study was performed in Imam Reza University Hospital Poison Center, (Mashhad, Iran) [13] from July 2012 to September 2012. Totally, 25 patients who were admitted to the hospital with Tramadol overdose and were confirmed to have ingested more than the recommended therapeutic dose (primarily by urinary test and secondarily by blood test) met the entry requirement. Among them, 11 patients consented to give at least two blood samples. Exclusion criteria were met and the treatment of patients [14,15] was not influenced by the process of data collecting. Ethics approval was obtained from ethics committee of the Mashhad University of Medical Sciences (MUMS/89/1876).

Blood samples, collected in heparinised glass tubes on admission and repeated later were investigated for Tramadol serum concentration using a high-performance liquid chromatographic system (HPLC) (Knauer, Germany) with the method explained in [16]. Intra- and interday variabilities were measured to determine the precision of the HPLC method. The relative standard deviation (RSD) of intraday and interday variations for Tramadol was 1.7% and 1.4% respectively, indicating that the method was repeatable. To measure the Half Life of Tramadol, first order kinetic (Formula: N_t_ = N_0_(1/2)^t/t1/2^) was assumed. Calculations were performed on blood concentration quantities. N_0_ denotes the first quantity, N_t_ is the quantity that is remained at the time of t and t_1/2_ denotes half-life.

All calculations were performed manually with the help of Microsoft® Office Excel 2003 and SPSS version 13.0 (SPSS Inc., Chicago, IL, USA).

### Findings

**Figure 1:**
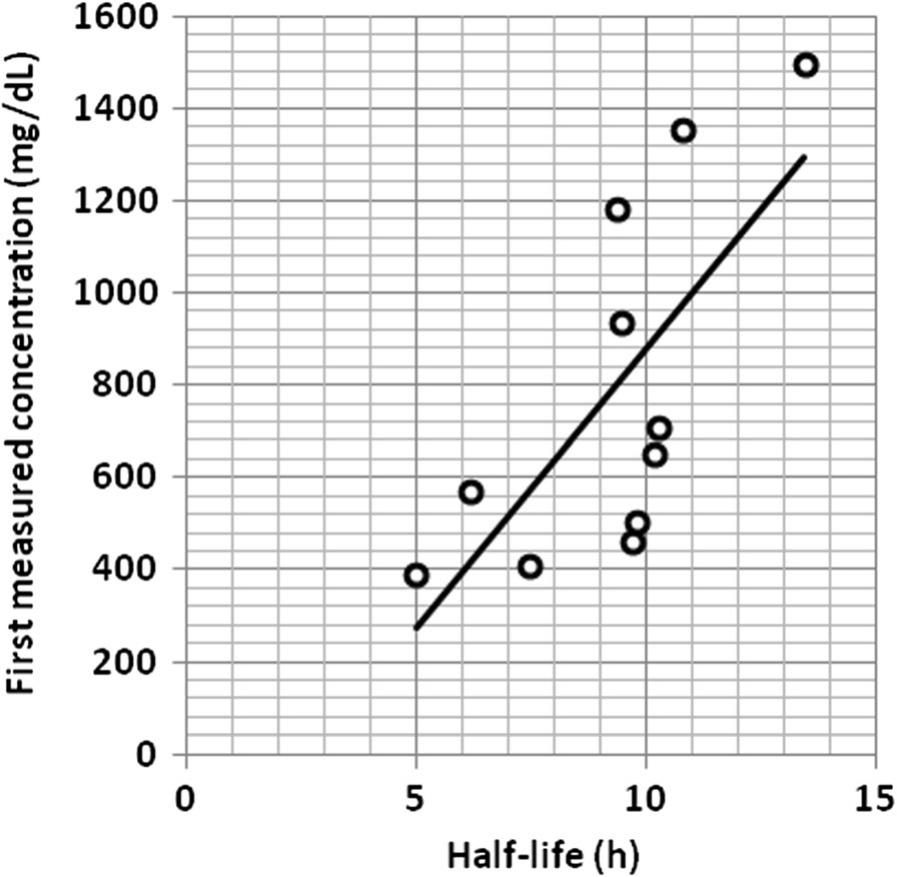
**Calculated Tramadol Half-life in relation to first measured blood concentration of Tramadol in overdose patients.** Half life is associated with higher concentrations (r = 0.708 Sig = 0.015).

Most of patients (9) were men (81.81%). Mean (Standard Deviation (SD), min-max) age was 23 years (8.142, 17–40). Mean (SD, min-max) first Tramadol concentration was 786.91 mg/dL (394.53, 391–1495). Mean (SD, min-max) second Tramadol concentration was 433.09 mg/dL (269.63, 148–950). Mean time interval from 1st to 2nd blood sampling was 8.23 hours (1.421, 5.5-11). Mean (SD, min-max) of Tramadol half life was calculated as 9.24 hours (2.310, 4.99-13.45). Half life was associated with higher concentrations (r = 0.708 Sig = 0.015) (Figure 1).

### Discussion

In this study, we found the half life of Tramadol to be 9.24 ± 2.310 hours in man in overdose which is comparable to that reported by Ardakani et al. [17]. They demonstrated the half life of Tramadol in healthy humans to be approximately 7 hours. Importantly, we demonstrated that Tramadol half life is dose dependent in overdose which may explain the further consequences of severe overdoses.

Tramadol is mostly metabolised by *O*- and *N*-demethylation and conjugation reactions which result in glucuronides and sulphates [18]. The wide variability in the pharmacokinetic properties of Tramadol is partly due to cytochrome P450 (CYP) polymorphism. In fact, Tramadol is mostly metabolized by the highly polymorphic enzyme CYP 2D6, meaning patients with different CYP2D6 genotypes may experience different degrees of pain relief and side effects [19,20]. To obtain more accurate results, we suggest that the toxicokinetic of Tramadol and its metabolites be studied in specific phenotypes. We also believe that in order to determine the definitive toxicokinetic profile of Tramadol, further investigations should be performed on larger groups.
